# 3D Reticulated Actuator Inspired by Plant Up-Righting Movement Through a Cortical Fiber Network

**DOI:** 10.3390/biomimetics6020033

**Published:** 2021-05-27

**Authors:** Tom Masselter, Olga Speck, Thomas Speck

**Affiliations:** 1 Plant Biomechanics Group @ Botanic Garden Freiburg, University of Freiburg, Schänzlestraße 1, D-79104 Freiburg, Germany; olga.speck@biologie.uni-freiburg.de (O.S.); thomas.speck@biologie.uni-freiburg.de (T.S.); 2 Freiburg Materials Research Center (FMF), University of Freiburg, Stefan-Meier-Straße 21, D-79104 Freiburg, Germany; 3 Cluster of Excellence livMatS @ FIT—Freiburg Center for Interactive Materials and Bioinspired Technologies, University of Freiburg, Georges-Köhler-Allee 105, D-79110 Freiburg, Germany

**Keywords:** actuator, bark, biomimetics, curving demonstrators

## Abstract

Since most plant movements take place through an interplay of elastic deformation and strengthening tissues, they are thus ideal concept generators for biomimetic hingeless actuators. In the framework of a biomimetic biology push process, we present the transfer of the functional movement principles of hollow tubular geometries that are surrounded by a net-like structure. Our plant models are the recent genera *Ochroma* (balsa) and *Carica* (papaya) as well as the fossil seed fern *Lyginopteris oldhamia*, which hold a net of macroscopic fiber structures enveloping the whole trunk. Asymmetries in these fiber nets, which are specifically caused by asymmetric growth of the secondary wood, enable the up-righting of inclined *Ochroma* and *Carica* stems. In a tubular net-like structure, the fiber angles play a crucial role in stress–strain relationships. When braided tubes are subjected to internal pressure, they become shorter and thicker if the fiber angle is greater than 54.7°. However, if the fiber angle is less than 54.7°, they become longer and thinner. In this article, we use straightforward functional demonstrators to show how insights into functional principles from living nature can be transferred into plant-inspired actuators with linear or asymmetric deformation.

## 1. Introduction

Most plants lack motility and are thus bound to their location. Nevertheless, many plants are capable of complex movements, such as the snapping action in both the carnivorous Venus flytrap and the water trap plant [1,2]. However, we often do not notice plant movements, either because the movements are too fast for the human eye, such as the trapping motion of the aquatic carnivorous bladderworts *Utricularia* spp. [3,4], or because plant motion takes place over a long period of time, such as the curving of branches caused by the formation of reaction wood in trees [5,6]. During biological evolution, plants have developed a variety of movement types and actuation mechanisms that enable either a rapid response to a stimulus or a long-term adaptation to changing environmental conditions and that provide great selective advantages for the survival of the respective plant species. Since most plant movements, in contrast to those of animals, take place without muscles and conventional (localized) hinges but through an interplay of elastic deformation and strengthening tissues, they are thus ideal concept generators for biomimetic hingeless actuators [7]. 

### 1.1. Movements of Plants

In multicellular plants, the duration of a movement is a function of the smallest macroscopic dimension of the moving part. Based on poroelastic time (=fastest tissue swelling) and inertial time (=fastest tissue elastic motion), the driving forces behind plant movements can be classified into hydraulic processes and mechanical instabilities. Hydraulically driven movements include irreversible growth processes and movement caused by reversible swelling or shrinking attributable to osmotic or humidity changes. Slow hydraulic movements can be speeded up by mechanical instabilities such as the release of stored elastic energy and rapid geometric changes [8,9]. 

Whereas some of the movements that occur with a change in humidity or turgor are dependent on the shape of the moving organ (cf. [2,9,10]), others are closely coupled to cylindrical geometries surrounded by a net-like structure [11,12]. An example of the latter is the structural setup enabling the movement of the feet of sea urchins [13,14]. 

### 1.2. Plant-Inspired Actuators

The transfer of principles of plant movements to biomimetic technical structures is challenging, as relatively few technical components are currently available that allow the implementation of hingeless elastic opening and closure and other functional aspects typical of plants. Nevertheless, biomimetic solutions to such technical problems show great potential and should lead to jump innovations. This also holds true for bio-inspired actuators that can be used in a wide range of technological applications including elastic systems in architecture, such as those achieved for the plant-inspired façade shading systems Flectofin and Flectofold (cf. [7,15]). In a biology push process (=biomimetic bottom-up approach), the Universities of Freiburg and Stuttgart have cooperated in the development of a hygroscopic demonstrator as a proof of concept for mimicking the opening and closure of pine cones [10,16]. Plant-inspired motion ideas have also been integrated in a hygroscopically actuated exhibit (Hygroscope, Centre Pompidou) and the Hygroskin pavilion at the FRAC center in Orléans [17,18].

Similar challenges have been faced within a biomimetic technology pull process (=biomimetic top-down approach) by [19] during the development of a plant-inspired actuator without articulated hinges on an architectural scale. The authors used the opening and closing movement of grass leaves as a model for the development of a biomimetic cellular actuator. Leaf halves of *Sesleria nitida* show pronounced kinetic amplification actuated by turgor changes of fan-shaped groups of bulliform cells that lie in the upper epidermis to the right and left of the leaf midrib. Sclerenchyma strands, together with vascular bundles, form strengthening spacers between the upper and lower epidermis and an elastic hinge-like structure at the midrib. The leaf halves fold shut when the turgor pressure in the bulliform cells is low and unfold again when the bulliform cells become fully turgescent. Based on this functional principle, Mader et al. [19] developed a pneumatic cellular actuator consisting of a row of single cells with compliant hinges positioned on a plate. When the pneumatic pressure applied to each individual cell is increased, the cell becomes wider along its upper side, and the entire structure bends. A first prototype was successfully integrated into the façade shading system Flectofold [20], in which the bending of the midrib controls the hoisting of its wings.

### 1.3. Conventional Technical Solutions

**Figure 1:**
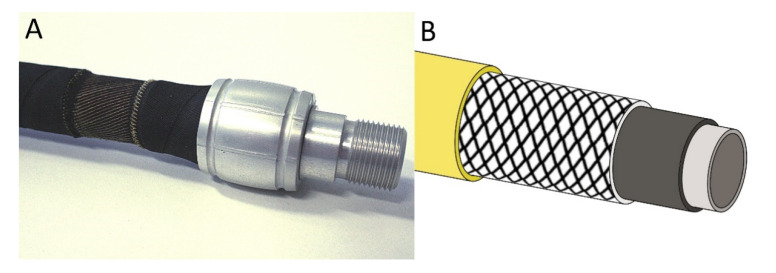
(**A**) The fluidic muscle (pneumatic muscle), which consists of a hollow elastomer cylinder with embedded aramid fibers, represents a tensile actuator. To make the fiber network visible, the outer coating was partially removed (© Festo AG & Co. KG, Esslingen, Germany). (**B**) Schematic drawing of a pressure-resistant hose with reinforcing fibers having an angle between the longitudinal axis and fibers of approximately 55°.

The movements of cross-linked net-like tubular tissues that are a distinct characteristic in many plants and animals (as described above) are in current technical use, e.g., in the so-called fluidic muscle that is produced by Festo AG & Co. KG (Figure 1) [21] and in which increasing air pressure leads to a shortening of the structure. By shortening these actuators, loads can be moved with a smooth motion pattern. The explanation of this principle of action has led to a popular science publication in which the mode of operation is illustrated with a simple experiment using balloons, commercially available vegetable packaging nets, and cable ties [22]. Nevertheless, we must not assume that the fluidic muscle is a biomimetic product. It represents an analogy of form and function in nature and technology, i.e., no transfer of knowledge occurred from a natural model to the technology involved in the development of the fluidic muscle. However, the combination of fluidic muscles enables complex actuation patterns such as bending and curving and is thus able to attain high degrees of freedom, as exhibited by the trunk of an elephant [23,24].

### 1.4. Aim of the Study 

**Figure 2:**
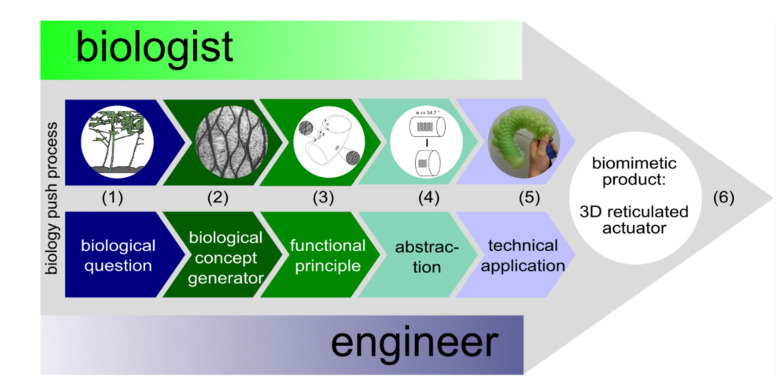
The biology push process (biomimetic bottom-up approach) of the curved 3D reticulated actuator. (**1**) What is the basis of the up-righting movement of inclined plant axes? (**2**) Morphological–anatomical investigations of *Lyginopteris oldhamia* revealed net-like structures in the cortex; (**3**) functional analysis of the actuation principle in stem up-righting (with permission of [12]); (**4**) length–volume relationships of cylinders depend on the fiber angle α between the spiral netted fibers and the longitudinal axis of the cylinder; (**5**) curved 3D reticulated demonstrator with asymmetric deformation: connecting the net meshes on the adaxial side and/or cutting the net meshes on the abaxial side result in a large curvature of the tube when inflated; (**6**) biomimetic product to be developed: 3D reticulated actuator for use in technology.

In this study, we aim to describe the development of a plant-inspired actuator that can be used to erect or bend rod-shaped objects. We describe the idea flow from the initial biological insights to various straightforward physical demonstrator models by using the biology push process (=biomimetic bottom-up approach) [25,26]. We present the six consecutive steps of the biomimetic development approach (Figure 2): (i) the biological question regarding net-like structures in plants, (ii) the selection and investigation of suitable models, (iii) the identification of the functional principle underlying the plant movement, (iv) the abstraction and translation into common language for natural scientists and engineers, (v) feasibility studies, and (vi) the outlook for possible applications in technology, education, and training.

## 2. Biology Push Process (Biomimetic Bottom-Up Approach)

### 2.1. Biological Question

**Figure 3:**
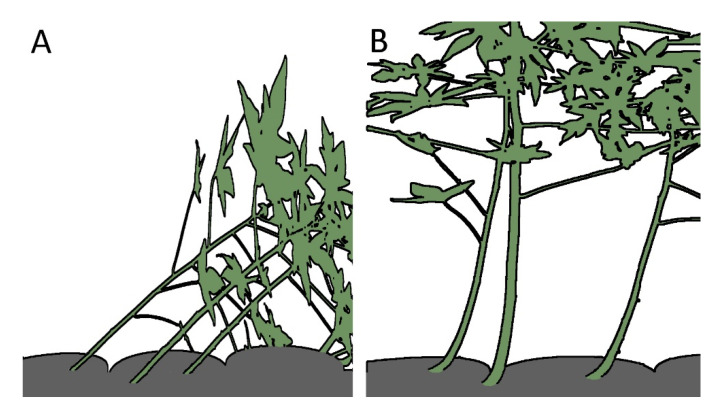
Erectile movement of *Carica papaya* stems. (**A**) Trees planted in tilted orientation with an inclination of 45°; (**B**) up-righting after 20 days resulting from active bending by growth of the stems mainly in the basal stem region. (**A**,**B**) redrawn and modified from [11].

The observation that tilted stems of papaya (*Carica papaya* L.; hereafter: *C. carica*) and leaning young trees of balsa (*Ochroma pyramidale* (Cav. ex Lam.) Urb.; hereafter: *O. pyramidale*) can reorient rapidly leads to the question of the underlying principles [11,27] (Figure 3). An understanding of the functional principle makes it all the more interesting, because structures similar to those of balsa and papaya have been found in *Lyginopteris oldhamia* (hereafter: *L. oldhamia*) and may provide information about the form–structure–function relationship in a fossil plant [12].

### 2.2. Biological Concept Generators

**Figure 4:**
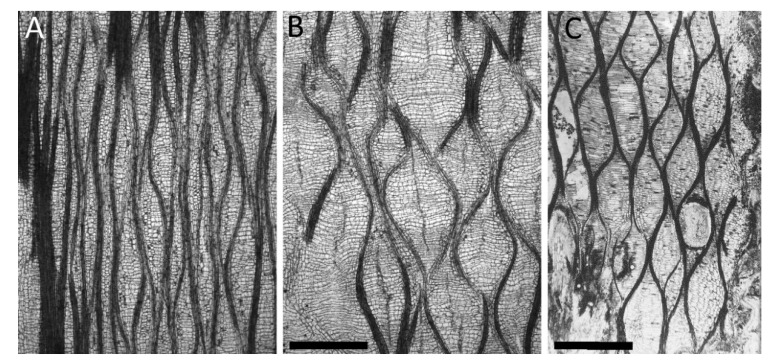
Tangential sections of the outer cortices of *Carica papaya* (**A**,**B**) and *Lyginopteris oldhamia* (**C**). (**A**) Unstrained abaxial side, (**B**) highly strained adaxial side, and (**C**) strained “Dictoxylon” cortex. Scale bars: (**A** = **B**) = 1 mm, (**C**) = 3 mm.

*C. carica* and *O. pyramidale* have in common that their outer cortex can be unequally strained because of asymmetric growth of their inner secondary tissues. However, they do not use fiber structures at the cell wall level, but instead employ, at a higher hierarchical tissue level, netted fiber structures enveloping the whole stem (Figure 4A,B).

In fossil records of *L. oldhamia*, an extinct species common in the Upper Carboniferous of Europe (300 Myr B.C.), we can find similar structures in the so-called “Dictyoxylon” outer cortex. The “Dictyoxylon” is believed to be able to stretch following the stresses imposed by the increase in volume of the inner secondary tissues [28,29]. Similar to the cortex structure found in *C. carica* and *O. pyramidale*, the “Dictyoxylon" cortex of *L. oldhamia* is composed of sclerenchymatous strands that are imbedded in a parenchymatous ground tissue and that form a net-like structure (Figure 4). Moreover, [12] have shown that asymmetric secondary growth can also be present in the fossil stems of *L. oldhamia*, and that straining of the cortex is higher in the cortex above zones of increased secondary growth (Figure 4C).

### 2.3. Functional Principle

**Figure 5:**
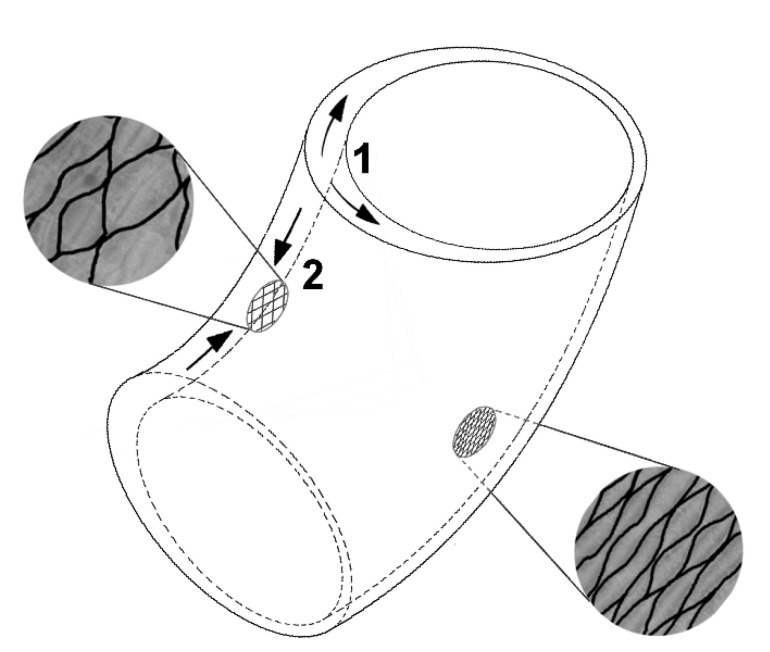
Erectile movement in *Carica papaya* and *Ochroma pyramidale* stems. Asymmetric growth in thickness of the secondary xylem creates stronger circumferential stresses (arrows 1) on the adaxial side of the stem (additional secondary wood formation); these stresses are partially converted into axial shortening (arrows 2) by the wrapping bark with its crossed net-like fiber bundles. Modified from [12].

Because of the unequal straining that occurs in the outer cortex and that is specifically caused by asymmetric secondary growth, the described extant plants are able to straighten inclined (non-vertical) stems (Figure 3) [27]. The asymmetric increase in volume of the vascular secondary tissues leads to higher straining (and thereby shortening) of the adaxial (upper) side than the abaxial (lower) side, resulting in a curvature of the axis (Figure 5). This functional principle is highly dependent on the angles of the net-like cortex structures in *C. papaya* and *O. pyramidale*. Because of structural similarities, we can even assume that the fossil plant *L. oldhamia* developed a comparable functional principle and was also able to straighten its axis [12,30].

### 2.4. Abstraction

To understand the functional principle of the biological concept generators described above, we first examined the simplified case of cylinders with braided fibers and equal straining before we proceeded to structures allowing for curvature.

**Figure 6:**
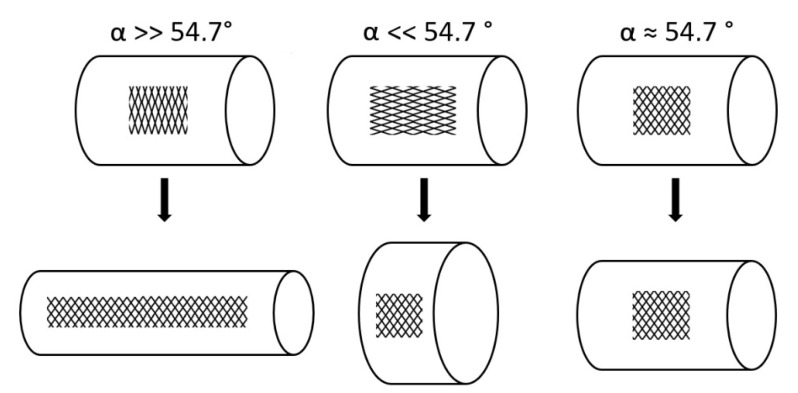
Length–volume relationships of cylinders surrounded by spiral netted fibers. The fiber angle α is the angle between the spiral netted fibers and the longitudinal axis of the cylinder. Maximal volume is reached when α = 54.7°. When the initial α is greater than 54.7°, the cylinder elongates and becomes thinner under internal pressure, whereas if the initial α is less than 54.7°, the cylinder shortens and thickens. At α = 54.7°, the length and volume remain constant under internal pressure.

Under internal pressure, the interaction of axial and (circumferential) tangential stresses causes braided cylinders with an angle α, between the longitudinal axis and the fibers, greater than 54.7° to lengthen and become thinner, while braided cylinders with an angle less than 54.7° shorten and thicken (Figure 6). An angle of 54.7° is the so-called neutral angle at which the volume under internal pressure remains constant (highest), and at which neither shortening nor elongation of the cylinder occurs [31]. 

**Figure 7:**
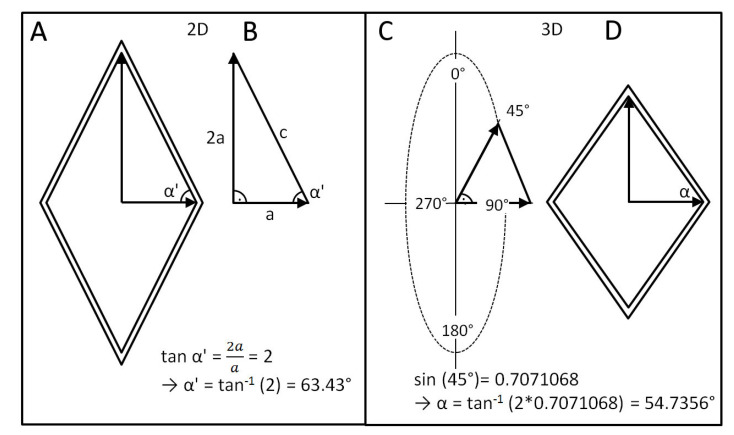
Geometric representations for determination of the neutral angle. (**A**,**B**) The angle α’ can be defined in a 2D case by the vector *a* (parallel to the longitudinal axis of the cylinder) and a vector 2*a* (vertical to the longitudinal axis of the cylinder) for a simplified two-dimensional case. (**C**,**D**) Taking into account the rotational axis, α’ can be transformed into the neutral angle α.

Why should this angle be 54.7°? In a cylinder that is pressurized from the inside and that is in a state of equilibrium, the circumferential stresses are always twice as high as the axial stresses [32], i.e., we can determine a theoretical angle α’ by abstracting the mesh in the cylinder, in a first step, to a two-dimensional right-angled triangle (=simplified force parallel diagram) (Figure 7A) with an edge length 2*a* (corresponding to the assumed vector of the circumferential stress) and a vertical edge length *a* (corresponding to the assumed vector of the axial stress) (Figure 7B). For this simplified two-dimensional mesh with twice as much stress in one direction as in another (perpendicular) direction, we obtain an angle of α’ = tan^−1^ (2) = 63.43°. 

However, in contrast to this simplified 2D model, the cylinder is a three-dimensional object, i.e., the simplified “2*a*/*a*” triangle is tilted on average by 45° to the two-dimensional plane. The value of 45° results as the mean value between the two extreme cases: (1) parallel to a 2D plane (i.e., tilted by 0 degrees) and (2) perpendicular to it (i.e., tilted by 90°). Thus, the real (3D) vector of circumferential stress is not 2*a* but 2*a* × sin 45° = 2*a* × 0.707 = 1.414, since the mesh is not in a flat plane but in a plane of rotation around the central longitudinal axis of the cylinder (Figure 7C). Therefore, the real neutral angle α = tan^−1^ (1.41*a*/*a*) = tan^−1^ (1.414) = 54.7° (Figure 7D).

This neutral angle is important, because it is used in many technical tubular structures in which radial expansion and axial contraction take place, such as pneumatic muscles reported by Festo [21] (Figure 2A), and other fluidic muscles [33]. The flexibility of the structure is also used in the so-called stents, which can be inserted into large blood vessels in order to avoid blockage. These stents are usually braided at an angle equal to or close to 54.7°. Equally important is the production of other tubes with mesh structures that are arranged at a neutral angle, e.g., in water-conducting hoses (Figure 2B). Here, shortening or expansion is undesirable; the aim is to enable the highest possible internal pressures without significant longitudinal/transverse deformation of the hose and to maintain volume. 

In *C. papaya*, *O. pyramidale*, and *L. oldhamia*, the angle α between the fibrous cortex structures and the longitudinal axis is significantly smaller than 54.7° (see Figure 4) so that a “pressurization,” i.e., stresses generated by the increase in volume of secondary tissues, leads to the shortening of the structure. Because of the asymmetry of the vascular secondary growth, the structure is only shortened on one side, and thereby, a curvature of the axes can be evoked and modified by the amount of asymmetric secondary growth.

### 2.5. Feasibility Studies

In this article, we will use the manufacturing of simple functional demonstrators to demonstrate the way in which insights into functional principles from living nature can be transferred into technical implementations. We will first reproduce the functional principle of linear shortening or elongation and then build a further developed (biomimetic) demonstrator with which a directional adjustment, i.e., bending and curving, can be achieved, as in the concept generators *O. pyramidale* and *C. papaya*.

#### 2.5.1. Technical Translation as Biomimetic Demonstrator with Linear Deformation

**Figure 8:**
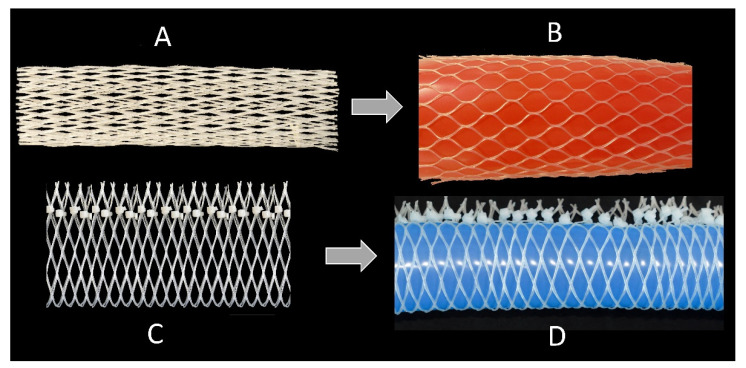
Linear mesh demonstrator. Change in the mesh geometry of bottle nets with an original angle considerably smaller than 54.7° (**A**), and considerably larger than 54.7° (**C**) and an additional fixation by cable ties that allows for lengthening. Inflation of the balloon leads to (**B**) a radial expansion and an axial shortening and (**D**) an axial elongation but no radial thinning.

We can demonstrate the deformation of the cross-linked netted tube structure in a simple experiment based on the experiments first published in [22]. For this simple proof of concept, we use inflatable elements (balloons) and net-like tube structures (so-called bottle nets). Inflation of the balloons in the bottle nets leads to a radial expansion and an axial shortening of the structure: we measured axial shortening to 90% of the original length and radial expansion to 125% of the original width, as the initial angle of the net fibers is 15° (i.e., much smaller than 54.7°) (Figure 8A,B). If, on the other hand, we cut open the bottle nets and connect them in the other direction by using cable ties, the angle is 90–15 = 75° and, theoretically, a radial thinning and axial expansion is expected. The measured axial elongation amounts to 30%, i.e., the structure elongates under internal pressure to 130% of its original length. However, no radial thinning is measurable (Figure 8C,D). The reasons that our simple experiment (proof of concept) does not work exactly as predicted by theory are as follows: (1) the balloons do not expand ideally in a barrel shape (but in a more spherical shape), despite the fibers being tied around them, (2) the nets do not form ideal rhomboid meshes but assume other patterns when they are deformed. We therefore do not find the postulated 54.7° mesh fiber angle but an approximation to it (compared with the angle of the initial structure) and (3) the net and the balloon are not firmly connected to each other at each end.

#### 2.5.2. Technical Translation as Biomimetic Demonstrator with Asymmetric Deformation

**Figure 9:**
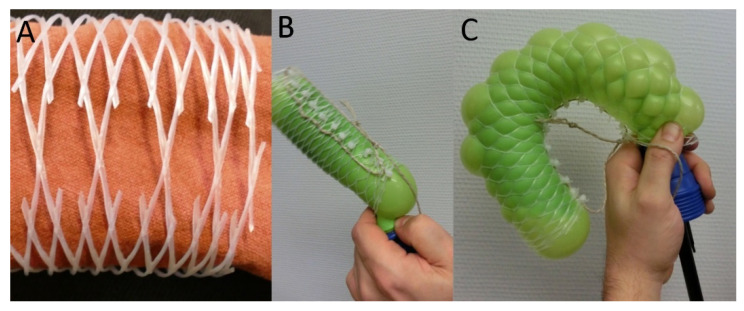
Curved mesh demonstrator. (**A**) Reduction of the resistance on the abaxial side by cutting out one rhombus in each repeat. (**B**) Connection of the net meshes on the adaxial side together with cutting each second mesh on the abaxial side results in (**C**) a pronounced curvature of the tube when inflated.

We created a special type of demonstrator by integrating a structural asymmetry into the tubular structure, following the example of the plants *O. pyramidale* and *C. papaya*. In the species mentioned, secondary thickness growth is asymmetric, causing a directional difference in internal pressure (see Section 2.2), as the structures expand more in the areas of increased tension caused by the increase in volume of the secondary tissues (see the description of the process above). Such a “directed” internal pressure is difficult to achieve with air-filled structures. Therefore, to produce a demonstrator with asymmetric expansion, the surrounding net-like structures have to be modified. We can adapt the bottle nets by obtaining larger meshes by cutting each second mesh on the adaxial side (here, rhombs are cut out as demonstrated in Figure 9A) so that they can expand more easily on the adaxial side than on the opposite side with the original denser meshing (the abaxial side; here, the meshes are additionally connected to each other by a household string to prevent expansion). A considerable curvature of the structure now occurs during inflation of the balloon (Figure 9B,C). The iterative functional principle of the technical demonstrator works in an “opposite” way to the natural models: initially uniform radial expansion results in a curvature as fewer fiber meshes, i.e., less opposing resistance, are present on one side “from the outset,” i.e., the asymmetrically arranged fibers cause the curvature. This is in contrast to the natural role models, in which the asymmetry of the fibers is a result of the directional secondary growth, i.e., an asymmetric increase in inner volume. Nevertheless, the result is similar: a curvature can be created. This simple example shows that, in bio-inspired implementation, both an understanding and an abstraction of the functional principle are important and are not the consequence of a 1:1 imitation.

## 3. Discussion

A comparison of the biological model structures and the bio-inspired technical demonstrators shows similarities and dissimilarities. Similarities arise from the abstraction of the functional principle and its translation into a common language for natural scientists and engineers. Differences arise because we do not use the biological model as a blueprint but, instead, have to reinterpret the functional principle for use in the technical field and produce the biomimetic product from technical materials. Moreover, we can even go beyond a single biological model and combine the functional principles of several models in the biomimetic product.

Similarities between the biological models and the technical application are as follows:

- *Structure:* The structural setup, i.e., a tubular geometry surrounded by a peripheral network of fibers, is similar in *C. papaya*, *O. pyramidale*, *L. oldhamia*, and the demonstrators of the bio-inspired 3D reticulated actuator.
- *Change of shape:* Length–volume relationship depends on the fiber angle α between the spiral netted fibers and the longitudinal axis of the cylinder: α=54.7° leads to a maximal volume, but, under internal pressure, the cylinder elongates and thins when α≫54.7° and shortens and thickens when α≪54.7°.

Dissimilarities between the biological models and the technical application are as follows:

- *Actuation:* Shortening/elongation of the cylindrical net structures is often hydraulically driven in nature, e.g., the swelling of the so-called G-layer [34] in cell walls of reaction (tension) wood on the upper side of branches in deciduous trees. The increase in volume of the cylindrical cellular structure that occurs here is converted into a shortening of the wood cells in the axial longitudinal direction by a special enveloping layer [5,6]. Thus, tension is generated in the tension wood of deciduous trees. An additional example of this actuation principle by liquids is the movement of the feet of sea urchins and starfish, in which extension or contraction is actuated by pressure changes in the ambulacral system [13]. On a different scale, the similar deformations of the netted fibers in *C. papaya*, *O. pyramidale*, and *L. oldhamia* are actuated by growth-induced stresses. All of these examples have in common that the actuation is markedly different from the pneumatic pressurization used in the demonstrators (main difference: incompressible versus compressible actuation agents, see below).
- *Speed of movement:* Re-orientation of plant stems takes place by slow growth-induced processes, both in tension wood and in cortex-based (re-)erection, compared with the rapid inflation-based size and shape change in the demonstrator. Nevertheless, the time frame of the movements achieved with the bio-inspired demonstrators above shows more similarities with that of the shape changes in the feet of sea urchins and starfish.
- *Reversibility:* Re-orientation in the plants is irrevocable, whereas the pressurization of the demonstrators can easily be undone; this again shows similarities with the reversible motion of the feet of sea urchins and starfish.
- *Power:* Forces exerted by liquids (turgor pressure, swelling of G-layers, liquid-filled feet of sea urchins) or solids (secondary tissues) are higher than the forces attainable by inflation because of the incompressibility of liquids and solids as compared with compressible air.
- *Curvature actuation:* As detailed above, curvature is the result of asymmetric growth in *C. papaya*, *O. pyramidale*, and *L. oldhamia*, whereas it is a consequence of the asymmetric net structure in the technical translation.

## 4. Conclusions and Outlook

Technical structures based on the functional principle described above might find application in the automotive industry and in aerospace, i.e., areas in which lightweight construction and adaptive shape adjustments are of particular interest. Such implementation is not necessarily limited to pneumatic structures: in the field of architecture, similar adaptive hulls can help to improve the mechanical properties of concrete sheathing by using FRP (fiber-reinforced composites). Here, the combination of bio-inspired braided structures [35,36,37,38] and the cross-linking presented in this study has a high potential for successful transfer. In addition, the above actuation can be developed and improved further by replacing the internal pressurized cylinder by reactive materials embedded in the meshes of the net-like hull, which isotropically or anisotropically swell or shrink upon external stimuli such as changes in humidity, pH, temperature, or light conditions. This would leave the entire inner volume of the 3D reticulated actuator free for additional integrated functions. In another implementation, the braided external structure could allow for various advantages in the spaces between the braided filaments, enabling transfer of light, gasses, and fluids in the absence of a continuous layer.

The described simple, easy-to-build, and inexpensive demonstrators not only can serve as a basis for technical implementations, but might also be well suited for teaching at university level and for education in high schools.

## Data Availability

Data is contained within the article.
